# New species of *Kudoa* **Meglitsch, 1947 (Cnidaria: Myxozoa)** infecting the swim bladder musculature of *Batrachoides surinamensis* (Bloch & Shneider) (Batracoidiformes: Batrachoididae)

**DOI:** 10.1007/s11230-026-10269-9

**Published:** 2026-04-06

**Authors:** Jhonata Eduard, Maria Eduarda Soares da Silva, Maria Alzirene de Souza Ferreira, José Ledamir Sindeaux-Neto, Michele Velasco, Evonnildo Costa Gonçalves

**Affiliations:** 1 1Morpho-Molecular Integration Laboratory and Technologies (LIMT), Institute of Animal Health and Production (ISPA), Federal Rural University of the Amazon (UFRA), Avenida Presidente Tancredo Neves, N◦ 2501 Bairro, Montese, Cidade, Belém, Pará Zip code: 66.077-901 Brazil; 2 Postgraduate Program in the Biology of Infectious and Parasitic Agents (PPGBAIP), Federal University of Pará (UFPA), Belem, Pará Brazil; 3 Postgraduate Program in Animal Health and Production in the Amazon (PPGSPAA), Federal Rural University of the Amazon (UFRA), Belém, Pará Brazil; 4 Postgraduate Program in Animal Reproduction in the Amazon (PPGREPROAMAZON), Federal Rural University of the Amazon (UFRA), Belém, Pará Brazil; 5 Biomolecular Technology Laboratory (LTB), Institute of Biological Sciences (ICB), Federal University of Pará (UFPA), Belem, Pará Brazil

**Keywords:** Multivalvulidae, Swim bladder, Fish parasite, Brazilian Amazon

## Abstract

The genus *Kudoa* is an important group of parasites within the class Myxozoa, as some species infect a wide range of fish hosts and cause post-mortem myoliquefaction, resulting in significant economic losses. This study describes a new species of *Kudoa* in toadfish *Batrachoides surinamensis*. In 60 % of specimens of *B. surinamensis* collected in the eastern Amazon, elongated white plasmodia were observed in the swim bladder muscle. Subquadrate myxospores measuring 5.6 µm in length, 7.5 µm in width, and 5.5 µm in thickness were recorded, with four polar capsules measuring 2.5 µm in length and 1.5 µm in width. Morphologically and morphometrically, these myxospores differ from those of other *Kudoa* species found in Amazonian fish. Histopathological analysis revealed that compression caused by plasmodia in muscle fibers, although no inflammatory infiltrates were observed. The Small subunit ribosomal (SSU) DNA sequence showed 97% similarity with a *p*-distance of 1.3% from *K. viseuensis*. In phylogeny, the new species formed a subclade with the species described in the Brazilian Amazon that infects fish from both marine and freshwater environments. The novel infection site, along with the morphological and molecular characteristics, supports the designation of a new species infecting the swim bladder musculature of *B. surinamensis*.

## Introduction

Myxozoa are parasitic cnidarians that account for approximately 18% of the diversity of the class, with most species infecting fish (Okamura et al., 2018; Whipps et al., 2026). Within this group, the subclass Myxosporea produces myxospores, which are essential for transmission between vertebrate hosts (typically fish) and invertebrate hosts (commonly annelids) (Lom & Dyková, 2006; Okamura et al., 2015; Alama-Bermejo & Holzer, 2021). The genus *Kudoa* Meglitsch, 1947, belonging to the order Multivalvulida, is characterized by star-shaped, subquadrate, or ovoid myxospores in apical view, containing internally four or more polar capsules and shell valves, with most species being histozoic where they form monosporous and polysporous trophozoites (Whipps et al., 2003). Most species cause skeletal musculature infections in fish; however, some species can also infect the brain, heart, ovaries, and organs of the gastrointestinal system, and may even cause systemic infections (Moran et al., 1999; Blaylock et al., 2004; Diamant et al., 2005; Wang et al., 2005; Burger & Adlard, 2011; Mansour et al., 2013; Velasco et al., 2015).

*Kudoa* spp. are distributed globally and have been reported in the Americas, Australia, Asia, Europe, and Africa (Eiras et al., 2014). In the Brazilian Amazon, eight species of *Kudoa* spp. have been described (*K. orbicularis*, *K. rousseauxii*, *K. amazonica*, *K. aequidens*, *K. ocellatus*, *K. viseuensis*, *K. yasai* and *K. ajurutellus*), infecting both freshwater and marine hosts (Azevedo et al. 2016; Cardim et al. 2020; Casal et al. 2008; Eiras et al. 2014; Monteiro et al. 2019; Neto et al. 2020; Silva et al. 2022; Velasco et al. 2019, 2022). The number of *Kudoa* species in this biome is still underestimated compared to the existing diversity of fish, which is considered one of the largest in the world (Azevedo et al., 2016; Velasco et al., 2019). Furthermore, fish are of socioeconomic importance to the local population (Casal et al., 2008).

The toadfish, *Batrachoides surinamensis* (Bloch & Schneider) is a carnivorous species of the Batrachoididae family that is distributed from Central America to the coast of Brazil. It lives in estuaries, preferring shallow, warm, and brackish waters with muddy bottoms, where it is highly adaptable to environmental changes (Barletta et al., 2003; Greenfield & Collette, 2008). Despite its wide distribution and ecological importance, information on its parasitic fauna remains scarce, especially concerning myxozoans.

In this context, the present study expands the knowledge of myxozoan diversity in this host by describing the morphological, histopathological, and molecular characteristics of a new species of *Kudoa* infecting the swim bladder muscles of *B. surinamensis* in the Brazilian Amazon. To our knowledge, this represents the first documented occurrence of this infection site in South America.

## Materials and Methods

### Collection of Fishes and Parasitological Examination

In Curuça, Pará State (0° 42′ 41″ S; 47° 52′ 33″ W), specimens of *B. surinamensis* (n=20), 10 of each sex (male and female), measuring 10.8±1.0 (7.0–16.2) cm in total length and weighing 15.5±3.0 (7.8 –28.7) g were collected dead (SISBIO license n. 86279/1) from fishing boats, then stored in isothermal boxes with ice, at an average temperature of 3°C, and transported to the Federal Rural University of the Amazon (UFRA), Belém Campus. The animals were necropsied at the Laboratory of Morphomolecular Integration and Technologies (LIMT). The presence of plasmodia was evaluated both on the body surface and internal organs using a light stereomicroscope, and the identification of *Kudoa* spp. was confirmed by visualizing the myxospores under an optical microscope.

### Morphological description

Fresh myxospores (n=30) were photographed in each plasmodium (n=2) of all infected fish under a differential interference contrast (DIC) microscope using AxioVision LE software version 4.8. The length, width, and thickness of the myxospores, as well as their internal structures (polar capsules), were measured (in µm) following the methodology established by Lom & Arthur (1989).

### Histopathological analysis

Fragments of swim bladders with confirmed infections were extracted for histological processing and preserved in Davidson’s solution (ethanol, formaldehyde, acetic acid, and distilled water) for 24 h. The samples were then dehydrated in an increasing ethanol sequence, diaphanized in xylol, and embedded in paraffin. Sections were cut at 5 µm thickness, and the slides were stained with both Hematoxylin-Eosin (HE) and Masson's Trichrome (MT) techniques (Luna, 1968).

### DNA extraction, PCR amplification, and sequencing

Fragments of bladder muscle tissue from five fish containing plasmodium were fixed in alcohol. DNA extraction was performed using the Purelink^TM^ Genomic DNA Kit (Invitrogen, ThermoFisher, CA, USA) following the manufacturer's instructions. The primer pairs ERIB1 (5′-ACCTGGTTGATCCTGCCAG-3′) and ERIB10 (5′-CTTCCGCAGGTTCACCTACGG-3′), followed by seminested ERIB10/MyxospecF (5′-TTCTGCCCTATCAACTWGTTG-3′) and ERIB1/MyxospecR (5′-GGTTTCNCDGRGGGMCCAAC-3′) were used for amplification of the SSU rDNA subunit (Barta et al., 1997; Bartošová et al., 2009). The polymerase chain reaction (PCR) was performed with a 25 µL solution containing 5-10 ng of DNA sample, 20 mM Tris (pH 8.4), 50 mM KCl, 4 mM dNTP (Invitrogen), 2 mM MgCl, and 1.2 units of Taq DNA polymerase (Invitrogen). The amplification process consisted of an initial denaturation at 95°C for 5 min, followed by 35 cycles of 95°C for 1 min, with annealing temperature at 50°C for 1 min and 72°C for 2 min, and finally 72°C for 5 min. In the second round, the same conditions were used, except that the annealing temperature was increased to 55°C. The samples were subjected to 1.5% agarose gel electrophoresis and purified with exonuclease I and alkaline phosphatase using the ExoProStar 1 Step kit (GE Healthcare, UK). Sample sequencing was performed with the AB 3,500 DNA automatic sequencer (Applied Biosystems™, Carlsbad, CA, USA) using BigDye^®^ Terminator v3.1, according to the manufacturer's specifications. The same PCR primer pairs were used to obtain amplicons for sequencing.

### Phylogenetic analysis

The small subunit ribosomal (SSU) rDNA consensus sequence was edited using BioEdit (Hall, 2011) and compared with sequences of other *Kudoa* spp. available in the National Center for Biotechnology Information GenBank database using the Basic Local Alignment Search Tool (BLASTn). The outgroup consisted of sequences from species of the genus *Unicapsula*: *U. seriloae* (LC577098), *U. andersanae* (KF184382), *U. pflugfelderi* (LC474131), *U. pyramidata* (AB971675), and *U. muscularis* (LC474141). The dataset containing *Kudoa* sequences and the outgroup sequences used for phylogenetic reconstruction was aligned with the MUSCLE algorithm implemented in AliView v.1.28 (Larsson, 2014). Maximum likelihood (ML) and Bayesian inference (BI) analyses were performed to reconstruct the phylogenetic tree. ML analysis was conducted using PhyML with 1,000 bootstrap replicates (Guindon et al. 2010). BI analysis was performed using MrBayes under the Markov chain Monte Carlo (MCMC) method for 5,000,000 generations; the first 1,000 trees were discarded as burn-in, and trees were sampled every 250 generations (Ronquist and Huelsenbeck 2003). The best-fit model of nucleotide substitution, selected using jModelTest v.2.1.10, was GTR+I+G. The genetic *p*-distance was calculated using PAUP version 5.0 (Wilgenbusch & Swofford, 2003).

## Results

### Morphological assessment

**Fig. 1.**
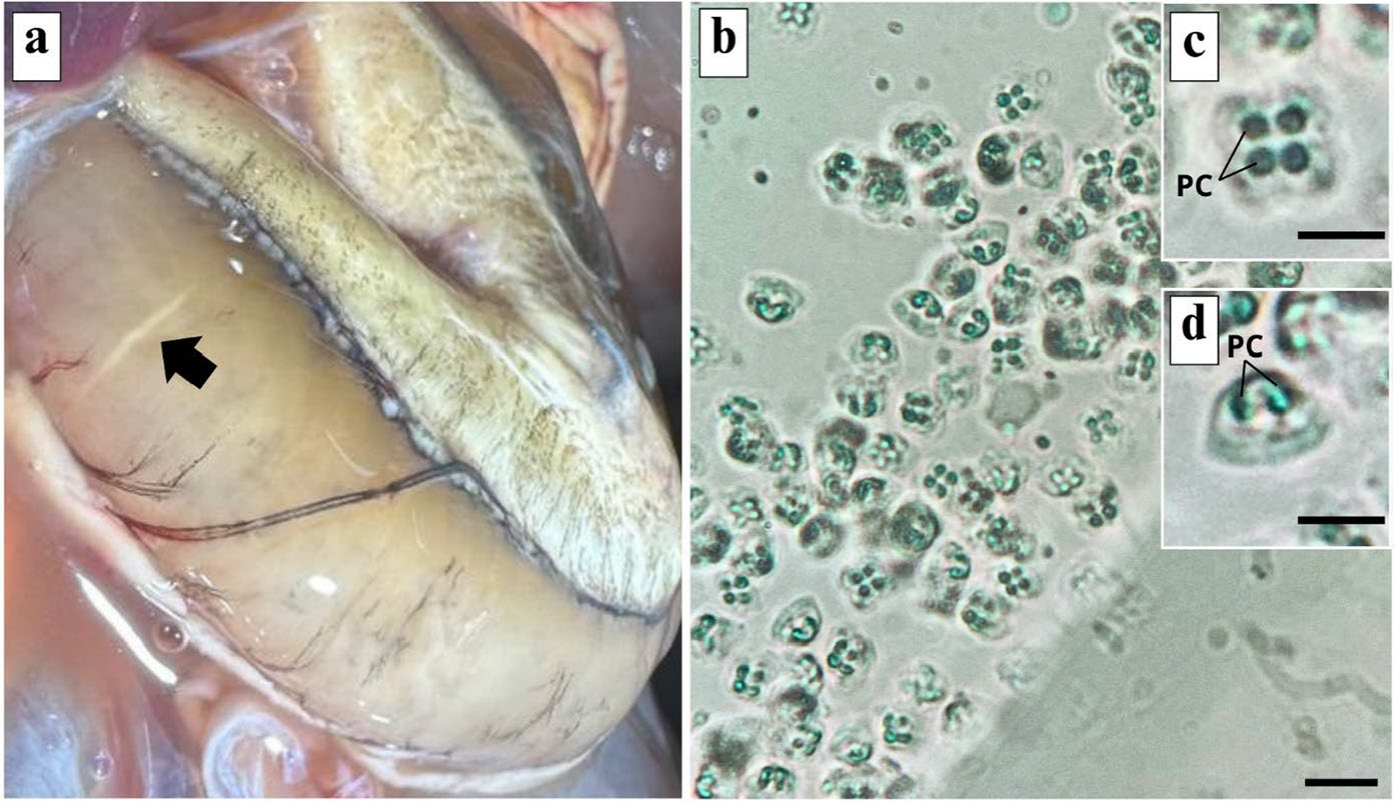
Microphotographs of ***Kudoa benignoi*** **n. sp.** infecting the swim bladder of the host *Batrachoides surinamensis*. **(a)** Plasmodium stage (arrow) embedded within muscle tissue, exhibiting a whitish appearance due to spore development. **(c)** Myxospores dispersed after the rupture of the plasmodium. Scale Bar: 10 µm. **(d)** Mature and fresh myxospores in valvar and sutural **(d)** view highlighting the polar capsules (PC). Scale bar: 5 µm.

Among the 20 specimens of *B. surinamensis* examined, 12 (60%) exhibited elongated, whitish plasmodia in the swim bladder musculature that were visible macroscopically (Fig. 1a). These plasmodia contained myxospores with morphological characteristics consistent with those of the genus *Kudoa* Meglitsch, 1947. Only infection by this type of myxosporean was observed in all specimens examined. Furthermore, no differences in prevalence were detected between males and females.

**Taxonomic Summary**

***Kudoa benignoi*** **n. sp.**

**Fig. 2.**
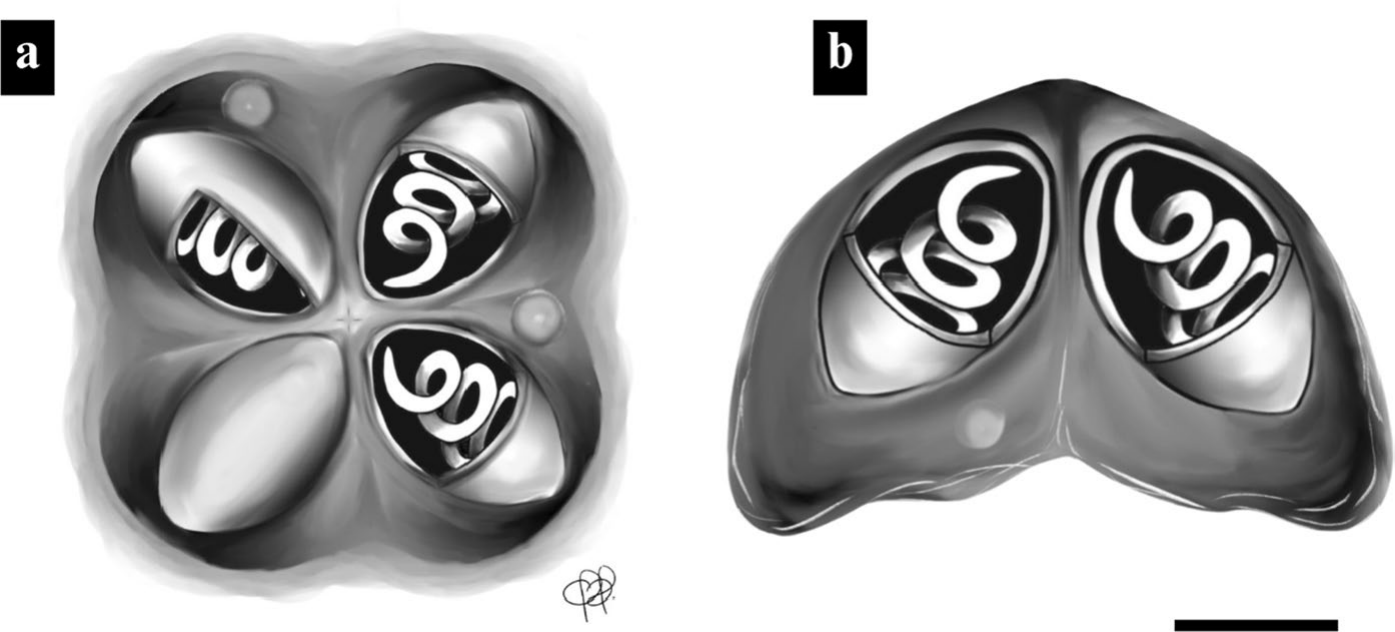
Schematic illustration of ***Kudoa benignoi* n. sp.** myxospores. (**a**) Apical view showing the arrangement of four equal-sized polar capsules (PC); (**b**) Lateral view demonstrating the myxospore shape. Scale bar: 2 µm.

**Description**: Myxospores in apical view were subquadrate with rounded valve ends, and in lateral view were pyramidal, measuring 5.6 ± 0.3 (5.3–5.8) µm in length, 7.5 ± 0.5 (6.5–7.8) µm in width, and 5.5 ± 0.6 (4.2–5.8) µm in thickness (Fig. 1b–d). The four polar capsules were elliptical and symmetrical, measuring 2.5 ± 0.2 (2.3–2.8) µm in length and 1.5 ± 0.1 (1.2–1.9) µm in width (Figs. 1c-d, 2a–b). The turns of the polar tubules were visible.

**Host:** *Batrachoides surinamensis* (Bloch & Schneider) (Batrachoidiformes, Batrachoididae).

**Type locality:** Curuçá, State of Pará, Brazil.

**Infection site:** Intercellular plasmodia in the striated muscles of the swim bladder.

**Prevalence:** 12/20 of the specimens analyzed (60%).

**Material deposited:** The histological slides from this study are registered in the Laboratory of Morphomolecular Integration and Technologies (LIMT) under the number GPSOA 180.

**Representative DNA sequences:** SSU rDNA sequences obtained from one plasmodium per *B. surinamensis* specimen showed less than 0.02% variation. The partial consensus sequence (1,449 bp) was deposited in the National Center for Biotechnology Information under accession number PQ849523.

**Etymology:** The species name honors Dr. Raimundo Nonato Moraes Benigno, an expert in animal parasitology at the Federal Rural University of the Amazon, Brazil.

### Histological analysis

**Fig. 3.**
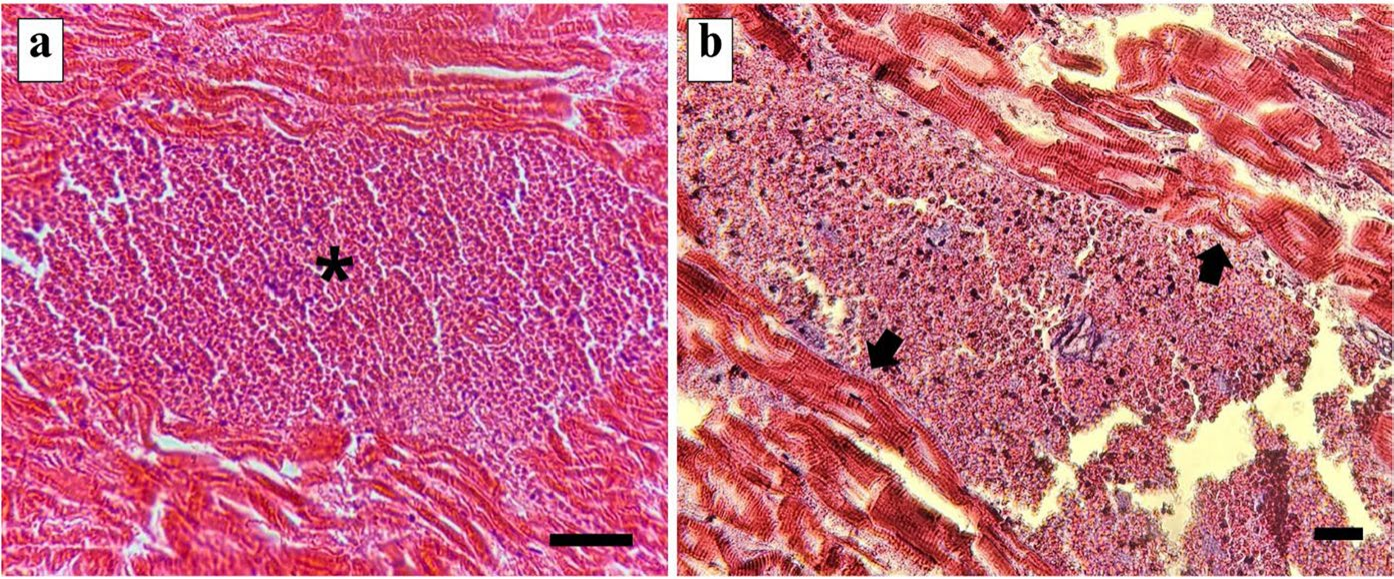
Histopathology of ***Kudoa benignoi* n. sp.** infection in the striated musculature of the *Batrachoides surinamensis* swim bladder. (**a**) Hematoxylin-eosin (HE) staining revealing intercellular plasmodia (*) developing within muscle fibers; (**b**) Masson’s trichrome (MT) staining showing released myxospores compressing adjacent myofibrils (arrow). Scale bar: 50 µm.

In histopathology plasmodia were identified within the intermuscular connective tissue of five specimens of *B. surinamensis*, located between adjacent skeletal muscle fibers rather than within the myofibers themselves (Fig. 3a). These alterations are compatible with mechanical compression and displacement of adjacent fibers caused by the expanding plasmodia (Fig. 3b).

### Phylogenetic analysis

In the BLAST analysis, the SSU rDNA sequence of ***Kudoa benignoi* n. sp.** (PQ849523) showed the highest identity with *K. viseuensis* (MK256272), found in the skeletal muscle of the same host, with 97% identity (1,356/1,396 nucleotides), including 15 insertion/deletion sites (indels). This was followed by *K. longichorda* (LC640010), which was isolated from the trunk muscle of *Decapterus tabl*, with 96% identity (1,391/1,451) and 12 indels. *K. parvibulbosa* (LC626079), isolated from the trunk muscle of *Megalaspis cordyla*, also exhibited 96% identity (1,390/1,451) with 12 indels.

**Fig. 4:**
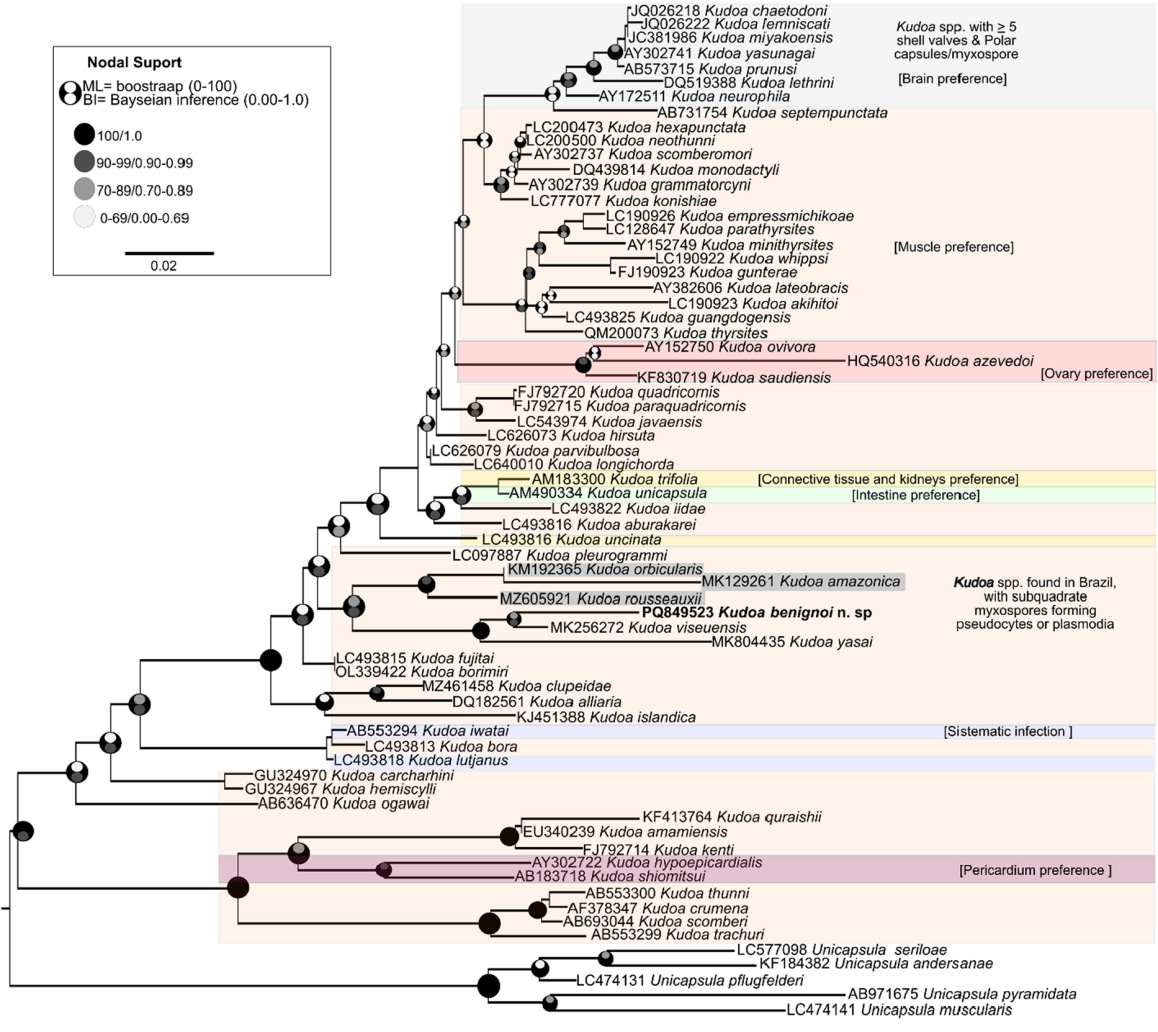
Maximum Likelihood (ML) concatenated phylogenetic tree of the SSU rDNA gene of *Kudoa* species, with their respective GenBank accession numbers, infection site and morphological aspects of the myxospores. Bootstrap support (ML) and posterior probability (BI) values ​​are shown. Species underlined in gray are those with freshwater habitat hosts.

In the phylogenetic analysis of SSU rDNA, the topology of the *Kudoa* tree was largely determined by morphological characteristics (such as myxospore shape and the number of sutural valves and polar capsules, host tropism, and habitat) (Fig. 4). The species *K. neurophila*, *K. lethrini*, *K. prunusi*, *K. yasunagai*, *K. miyakoensis*, *K. lemniscati*, and *K. chaetodoni*, all of which infect the nervous system, formed a monophyletic group characterized by the presence of five or more polar capsules (PC) and shell valves (SV). Additionally, *K. ovipora*, *K. azevedoi*, and *K. saudiensis* were closely related due to their common infection of the host's ovary. Similarly, *K. hypoepicardialis* and its sister species *K. shiomitsui* exhibited shared tropism for the pericardium system.

***K. benignoi* n. sp.** clustered in a well-supported subclade with other species described in fish from the Brazilian Amazon. These species, which have square or subquadrate myxospores and form pseudocysts in the esophageal musculature (such as *K. amazonica* in *Hypophthalmus marginatus*) or skeletal musculature (such as *K. viseuensis* in *B. surinamensis*, *K. yasai* in *Macrodon ancylodon*, *K. orbicularis* in *Chaetobranchopsis orbicularis*, and *K. rousseauxii* in *Brachyplatystoma rousseauxii*), shared common features with ***K. benignoi* n. sp.** Notably, ***K. benignoi* n. sp.** was part of the largest clade consisting of species from marine habitat

## Discussion

Members of the Batrachoididae family are distributed throughout South America and Central America and comprise 23 genera and 83 species (Greenfield & Collette, 2008). However, relatively few myxozoan species have been described in these hosts, with only two species reported infecting *Batrachoides surinamensis*: *Ortholinea abadiensis* Eduard et al. (2025), found in the urinary bladder, and *Kudoa viseuensis* Monteiro et al. (2019), infecting the trunk skeletal musculature. Therefore, ***K*.* benignoi* n. sp.** represents the third myxozoan species described in this host and the first reported infecting the swim bladder musculature.

Despite sharing the same host, ***K. benignoi* n. sp.** differs consistently from *K. viseuensis*, supporting their recognition as distinct species. First, the collection sites are separated by approximately 194.34 km, suggesting geographic segregation. Furthermore, the species differ markedly in infection site and plasmodial development. *K. viseuensis* forms intracellular plasmodia within the skeletal musculature of trunk, clearly delimited by connective tissue fibers, whereas ***K. benignoi* n. sp.** presents less well-defined interfibrillar plasmodia in the swim bladder. Morphological and morphometric differences in the myxospores further support this distinction. The myxospores of *K. viseuensis*, originally described as pseudoquadrate and later interpreted as subquadrate according to Whipps et al. (2003), exhibit more rounded valve edges, smaller width (7.2 *vs* 7.5 µm) and length (5.2 *vs* 5.5 µm), and relatively longer and narrower polar capsules (2.7 *vs* 2.5 µm; 1.2 *vs*1.5 µm) compared with those of ***K. benignoi* n. sp.**

Histopathological analysis revealed compression of muscle fibers caused by the development of polysporous plasmodia. However, no inflammatory response was observed following plasmodial rupture, in contrast to previous studies in which the release of myxospores into the interstitial spaces of the muscle induced inflammation, muscular atrophy, and necrosis, subsequently leading to post-mortem myoliquefaction due to the release of proteolytic enzymes (Ibrahim et al., 2024; Moran et al., 1999; Woodyard et al., 2022). The absence of an inflammatory response in the present study may be associated with several factors, such as storage temperature, time, and host immune response (Huda et al., 2025; Samaranayaka et al., 2006).

In the phylogenetic analysis, ***Kudoa benignoi* n. sp.** was positioned within a lineage composed of *Kudoa* species characterized by subquadrate myxospores with four SVs/PCs. This pattern is consistent with phylogenies based on the SSU rDNA gene, in which spore morphotype has been shown to be more determinant for clade formation than characteristics such as infection site tropism and host family (Li et al., 2024; Shin et al., 2016). ***Kudoa benignoi* n. sp.** formed a clade with *Kudoa* spp. described from marine fishes occurring in estuarine environments, particularly *K. viseuensis* and *K. yasai*, showing genetic distances of 1.3% and 3.4%, respectively. Similarly low levels of nucleotide variation have also been reported in molecular analyses of other *Kudoa* species, including *K. empressmichikoae*, *K. akihitoi*, and *K. cookii* (Heiniger et al., 2013; Kasai et al., 2016). This genetic similarity is likely associated with the high conservation of ribosomal DNA genes, including LSU rDNA, which evolve slowly (Kasai et al., 2016). Additionally, this pattern may be related to gene flow among these species, a key factor influencing genetic variation. The absence of natural barriers limiting dispersal may facilitate genetic connectivity, thereby reducing genetic differentiation and limiting the time required for complete speciation to occur (Kasai et al., 2016; Whipps & Kent, 2006).

In summary, ***K. benignoi* n. sp.** has an infection site not yet reported for *Kudoa* species in South America. Its phylogenetic position on a more basal branch suggests that freshwater *Kudoa* species in South America are derived from marine environments. The description of this new species contributes to the systematics of myxozoans in fish of the order Batrachoidiformes, especially *Batrachoides surinamensis*, a species commonly sold at local markets.

## Data Availability

The histological slides from this study are registered in the Laboratory of Morphomolecular Integration and Technologies (LIMT) under the number GPSOA 180, and the SSU rDNA sequence can be accessed in the nucleotide sequence database under the code number PQ849523.
